# Evaluating the Effects of Nusinersen Treatment in Adults With Spinal Muscular Atrophy Using Axonal Excitability and MscanFit MUNE

**DOI:** 10.1002/mus.28476

**Published:** 2025-07-24

**Authors:** Abir Alaamel, Nazan Şimşek Erdem, Gökçe Yağmur Güneş Gencer, Hüseyin Can Kaya, Hilmi Uysal

**Affiliations:** ^1^ Faculty of Medicine, Department of Neurology Akdeniz University Antalya Türkiye; ^2^ Department of Neurology Private ASV Yasam Hospital Antalya Türkiye; ^3^ Faculty of Health Sciences Akdeniz University Antalya Türkiye

**Keywords:** adult SMA, axonal excitability, MScanFit MUNE, neurophysiology, nusinersen

## Abstract

**Introduction/Aims:**

The biological changes in motor neurons and motor axons that correlate with the clinical benefits of nusinersen, an antisense oligonucleotide, in spinal muscular atrophy (SMA) remain poorly understood. This study aimed to investigate changes in axonal excitability and motor unit number estimation (MUNE) parameters following a four‐dose loading regimen of nusinersen in adult SMA patients.

**Methods:**

Adult patients with SMA were assessed using the Hammersmith Functional Motor Scale Expanded (HFMSE) and the Medical Research Council (MRC) scale at baseline and after nusinersen treatment. Axonal excitability studies and MScanFit MUNE were conducted in SMA patients before and after treatment. Baseline axonal excitability and MScanFit MUNE parameters in SMA patients were compared with those of a healthy control (HC) group.

**Results:**

Compared to the HC group (*n* = 10), SMA patients (*n* = 12) exhibited a significantly prolonged strength‐duration time constant (SDTC), a higher resting current/voltage (I/V) slope, and prolonged refractoriness at 2.5 ms. However, no significant changes in axonal excitability parameters were observed following nusinersen treatment. Similarly, there were no significant changes in MUNE or in other parameters, including D50, compound muscle action potentials, and steps%. In contrast, a significant increase in HFMSE and MRC scores was observed after treatment (*p* < 0.01 and *p* = 0.01, respectively).

**Discussion:**

A prolongation of SDTC, likely due to its effect on sodium channel function, was observed in this study, consistent with existing literature. Despite improvements in motor function, no significant electrophysiological changes were detected in adult SMA patients.

**Abbreviations**
CMAP: Compound muscle action potential
HFMSE: Hammersmith functional motor scale expanded
I/V: Current–voltage relationship
MRC: Medical research council
MUNE: Motor unit number estimation
RC: Recovery cycle
RRP: Relative refractory period
SDTC: Strength duration‐time constant
SMA: Spinal muscular atrophy
SMN2: Survival motor neuron 2
TE: Threshold electrotonus

## 1 Introduction

Nusinersen, an antisense oligonucleotide, is a disease‐modifying therapy that modulates the splicing of the survival motor neuron 2 (*SMN2*) gene to increase the production of normal, full‐length survival motor neuron protein [1]. An increasing number of studies in the literature report the safety and efficacy of nusinersen treatment in improving motor function in adult patients with spinal muscular atrophy (SMA) [2, 3, 4, 5]. Nevertheless, evidence regarding the efficacy of nusinersen therapy in these patients remains limited, especially when evaluated through objective neurophysiological assessments.

Axonal excitability measures have been utilized to understand the dysfunction of axonal conductance and passive membrane properties in patients with SMA [6]. Also, axonal excitability changes have been monitored in children with SMA treated with nusinersen. Nusinersen treatment was reported to facilitate the developmental recovery of motor axons and reduce degeneration [7]. MScanFit is an electrophysiological motor unit number estimation (MUNE) method to quantify the degree of axonal loss and compensatory reinnervation and the remaining number of functioning muscle fibers. The MScanFit technique has also been used to monitor the response to nusinersen therapy in pediatric and adult SMA patients [8].

This study aimed to investigate alterations in axonal excitability and MScanFit parameters following the four‐dose loading nusinersen treatment in adult patients with SMA.

## 2 Methods

This study was approved by the Akdeniz University Local Ethics Committee. Written informed consent was obtained from all participants. Patients with a confirmed genetic and clinical diagnosis of SMA who had received the four‐dose loading regimen of intrathecal nusinersen were included. Exclusion criteria encompassed patients requiring invasive mechanical ventilation, those with severe atrophy of the abductor pollicis brevis (APB) muscle, diabetes, chronic kidney disease, or other systemic disorders. All patients received 12 mg of nusinersen on Days 1, 29, 85, and 274, following the CHERISH [9] study protocol and national policies. Patients with SMA were evaluated at the initiation of nusinersen therapy and during the first maintenance phase (15th month) of the dosing regimen. Clinical and motor functions were assessed using the Hammersmith Functional Motor Scale Expanded (HFMSE) [10]. Muscle strength in patients with SMA was assessed using the Medical Research Council (MRC) scale [11]. The MRC sum score comprises the MRC scores for neck flexion/extension, bilateral shoulder abduction, elbow flexion/extension, wrist flexion/extension, finger flexion/extension, hip flexion, knee flexion/extension, ankle dorsiflexion/plantar flexion, and hallux flexion/extension, with a maximum normal strength score of 150.

Axonal excitability studies and MScanFit MUNE were performed in patients with SMA both before nusinersen treatment and during the first maintenance phase (15th month) of the dosing regimen. Additionally, pre‐treatment axonal excitability and MScanFit MUNE parameters in SMA patients were compared with those of age‐matched healthy controls (HCs) with no systemic or neurological disorders.

### 2.1 Axonal Excitability Assessment

Axonal excitability of the median nerve was assessed using the “TRONDF” protocol in QtracS software (Hugh Bostock, Institute of Neurology, London). In this protocol, 40% of the supramaximal response was selected as the target amplitude after evaluating the stimulation response. The active electrode was placed over the APB muscle and the reference electrode positioned on the proximal phalanx of the thumb. The median nerve was stimulated at the wrist. The following axonal excitability parameters were recorded: stimulus–response curves (SR), threshold electrotonus (TE), strength‐duration time constant (SDTC), current/threshold relationship (I/V), and recovery cycle (RC) (Table S1) [12].

### 2.2 MScanFit Assessment

The MScanFit MUNE was evaluated using QtracS software. The active recording electrode was placed over the APB muscle on the better‐functioning hand, while the reference electrode was positioned on the interphalangeal joint of the thumb. The median nerve was stimulated at the wrist, with the stimulation intensity manually increased until supramaximal stimulation was achieved. Then, the stimulus intensity was automatically decreased at a frequency of 1 Hz in 0.2% steps until no motor response remained. The MUNE, D50 (representing the number of largest consecutive differences required to generate 50% of the maximum compound muscle action potentials [CMAP]), and step% values were determined.

### 2.3 Statistical Analysis

Statistical analyses were conducted using the QtracP program and SPSS version 21 (IBM, Armonk, NY). Axonal excitability and MScanFit MUNE statistics were analyzed using the embedded program in QtracP, which automatically selects the most appropriate test based on Lilliefors' test for normality. Paired *t*‐tests were used to compare axonal excitability data before and after nusinersen treatment in SMA patients, while unpaired *t*‐tests were used for comparisons between the control and SMA groups. For MScanFit parameter analysis in the SMA group, paired comparisons were performed using the Wilcoxon signed‐rank test. Comparisons of MScan parameters between healthy controls and SMA patients were conducted using an unpaired *t*‐test. The MUNE, MRC scale, and HFMSE scores were analyzed using the Wilcoxon signed‐rank test for pre‐ and post‐treatment comparisons in SMA patients. A *p*‐value of less than 0.05 was considered statistically significant.

## 3 Results

**FIGURE 1:**
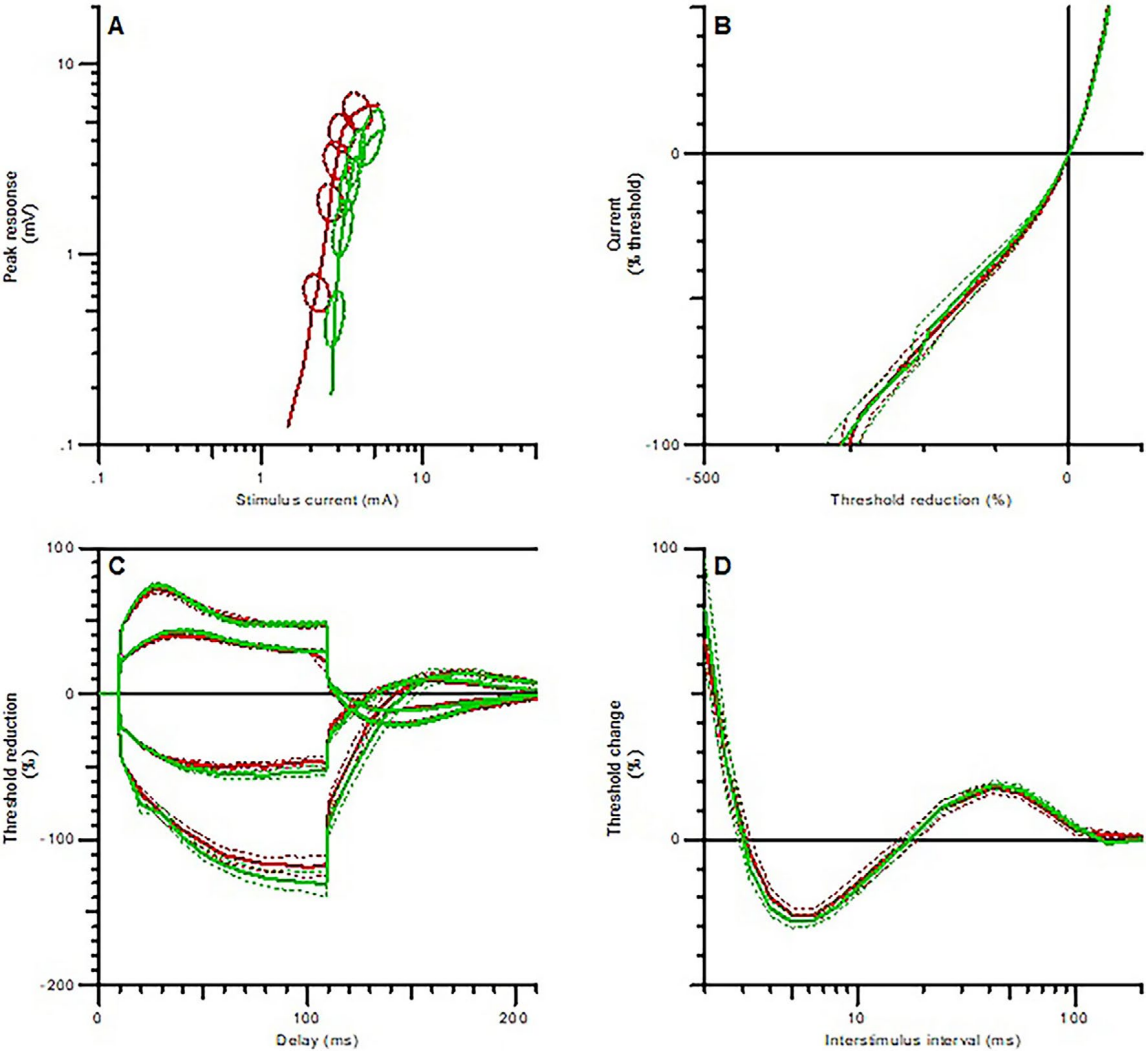
Axonal excitability parameters of adult SMA patients. Pre‐treatment (red) and post‐treatment (green). The dashed lines indicate standard error of mean (SE). A) stimulus‐response curve, B) current‐voltage relationship, C) threshold electrotonus, and D) recovery cycle.

Axonal excitability studies were performed on twelve adults with SMA type 2 (*n* = 3) and SMA type 3 (*n* = 9), with a mean age of 32.75 ± 2.85 years. The SDTC was significantly longer in SMA patients than in the HCs. The resting I/V slope was also higher in SMA patients compared to the HCs. Additionally, refractoriness at 2.5 ms was prolonged in the SMA group relative to the HCs (Table 1). No significant differences were observed in axonal excitability parameters before and after nusinersen treatment (Figure 1).

**TABLE 1:** Axonal excitability parameters of SMA patients pre‐post treatment with nusinersen and healthy controls.

	Mean ± SD			*p*	
	Healthy controls (HC) (*n* = 10)	SMA pre‐treatment (*n* = 12)	SMA post‐treatment (*n* = 12)	*p* pre‐treatment SMA vs. HCs	*p* SMA pre‐treatment vs. post‐treatment
TEh (90–100 ms)	−135 ± 5.9	−118.5 ± 6.9	−130.1 ± 7.6	0.08	0.27
Resting I/V slope	0.51 ± 0.02	0.65 ± 0.05	0.58 ± 0.02	**0.03**	0.27
TEh (20–40 ms)	−99.04 ± 2.3	−93.11 ± 4.3	−97.17 ± 4.1	0.27	0.51
RRP (ms)	2.61 ± 1.04	3.05 ± 1.05	2.91 ± 1.05	**0.01**	0.49
Refractoriness at 2.5 ms	5.43 ± 5.2	26.53 ± 7.8	26.56 ± 10.4	**0.04**	0.94
Minimum I/V slope	0.23 ± 0.01	0.25 ± 0.01	0.25 ± 0.02	0.60	0.79
SDTC	0.40 ± 0.01	0.52 ± 0.04	0.50 ± 0.02	**0.02**	0.75
Stimulus (mA) for 50% m	3.40 ± 1.09	2.92 ± 1.1	3.49 ± 1.12	0.40	0.35
Temperature (°C)	32.56 ± 0.59	33 ± 0.2	32.82 ± 0.26	0.48	0.60

*Note:* Bold fonts represent *p* < 0.05 values.

Abbreviations: I/V, voltage–current relationship; RRP, relative refractory period; SDTC, strength duration‐time constant; TEh, threshold electrotonus.

MScanFit MUNE analysis was performed in nine patients. The motor unit number in the HCs (117.0 ± 31.47) was significantly higher than in SMA patients (30.56 ± 14.93) (*p* < 0.001). Similarly, the CMAP amplitude was significantly higher in the HCs (11.09 ± 0.95) than in SMA patients (5.75 ± 0.78) (*p* < 0.001). The MScSteps% was significantly lower in the HCs (9.60 ± 1.89) compared to SMA patients (29.99 ± 6.90) (*p* = 0.01). No significant changes were observed in the MUNE values and size parameters of SMA patients after treatment, nor were there significant differences in other parameters, including D50, CMAP, and steps% (Table 2). A significant increase in HFMSE and MRC scores was observed following nusinersen treatment (Table 2).

**TABLE 2:** MScan and clinical parameters of SMA patients before and after treatment with nusinersen.

	SMA pre‐treatment (*N* = 9), mean ± SD	SMA post‐treatment (*N* = 9), mean ± SD	*p*
HFMSE	22.0 ± 20.5	26.58 ± 21.1	**0.008**
MRC	89.75 ± 36.2	94.0 ± 38.2	**0.01**
MUNE	30.5 ± 14.9	23.4 ± 10.8	0.1
CMAPmax	5.75 ± 0.8	6.05 ± 0.9	0.7
MScD50	28.67 ± 9.6	19.44 ± 5.3	0.7
MScSteps (%)	29.99 ± 7.2	38.54 ± 7.7	0.43

*Note:* Bold fonts represent *p* < 0.05 values.

Abbreviations: CMAPmax, Maximal compound muscle action potential; HFMSE, Hammersmith Functional Motor Scale Expanded; MRC, Medical Research Council; MUNE, motor unit number estimation.

## 4 Discussion

Consistent with previous research, our findings indicate that SMA patients exhibited prolonged SDTC compared to HCs. This finding is associated with dysfunction in axonal sodium conductance, as well as alterations in passive membrane properties [6].

A previous study reported no significant difference in the RRP (relative refractory period) between SMA patients and HCs [6]. However, our study demonstrated a prolongation of the RRP and the refractory period at 2.5 ms in the SMA group compared to HCs. These findings may indicate a potential involvement of sodium channel inactivation mechanisms.

In our study, axonal excitability parameters remained unchanged following nusinersen treatment in adult SMA patients. In contrast, Kariyawasam et al. investigated axonal excitability changes in pediatric SMA patients after nusinersen therapy and reported an increase in CMAP amplitude and resting current‐threshold slope, along with a reduction in axonal threshold and subexcitability after the loading doses. They concluded that nusinersen therapy enhances the developmental trajectory of motor axons and mitigates degeneration, particularly in children who received early treatment [7].

This study found no significant changes in MUNE values and size parameters, CMAP amplitude, or D50 values following nusinersen treatment. Kariyawasam et al. found that MUNE and D50 values increased in a cohort of 20 child patients with SMA following intrathecal nusinersen treatment [13]. Schneider et al. found no significant differences in the analysis of MScanFit variables following nusinersen treatment in the overall adult SMA patient group. However, an improvement in the number of motor units was observed only in the subgroup of SMA patients with the ability to walk [14]. The lack of changes in axonal excitability and MScanFit values in this study may be attributed to the older age, longer disease duration, and delayed onset of treatment in our cohort. Improvement in motor functions following treatment may be influenced by multiple factors, including concurrent physical therapy, patient motivation, and potential placebo effects. As national policy in Türkiye requires a minimum 3‐point increase in HFMSE scores to continue nusinersen maintenance therapy, all patients made considerable efforts to meet this criterion, including participating in regular physical therapy and rehabilitation.

A standardized procedure was employed to assess muscle strength; however, the absence of an objective measurement tool represents a key limitation of the study. Moreover, the single‐center design, along with the homogeneity of the study population in terms of ethnicity and geographical background, limits the generalizability of the findings. The relatively small sample size—particularly the low number of patients with SMA type 2—constitutes another limitation.

## 5 Conclusion

Our study demonstrated no significant changes in axonal excitability or MScanFit parameters following nusinersen therapy, despite the observed improvement in motor function in adult SMA patients.

## Author Contributions

**Abir Alaamel:** writing – original draft, writing – review and editing, software, data curation, methodology. **Nazan Şimşek Erdem:** writing – original draft, writing – review and editing, data curation. **Gökçe Yağmur Güneş Gencer:** writing – review and editing, data curation. **Hüseyin Can Kaya:** data curation. **Hilmi Uysal:** supervision, methodology, writing – review and editing, writing – original draft, project administration.

## Conflicts of Interest

The authors declare no conflicts of interest.

## Supporting information


**Table S1:** Axonal excitability parameters.

## Data Availability

Research data are not shared.
