# Supplementary material for: Evaluating the Effects of Nusinersen Treatment in Adults With Spinal Muscular Atrophy Using Axonal Excitability and MscanFit MUNE
Source: Muscle Nerve. 2025 Jul 24;72(3):515–9. doi: 10.1002/mus.28476 (PMC12338010; doi:10.1002/mus.28476)
Supplement: Supplementary file 1 — Table S1: Axonal excitability parameters. [file MUS-72-515-s001.docx]

**Supplementary Table 1.** Axonal excitability parameters

| Stimulus response curve (SR) | Curve showing the relationship between stimulus and response. |
| --- | --- |
| Strength duration time constant (SDTC) | The shortest stimulus duration time required for a stimulus twice the rheobase to evoke an action potential. |
| Threshold electrotonus (TE) | The change in excitability caused by a subthreshold current pulse |
| Current-voltage relationship (I/V) | The change in threshold that occurs at the end of the hyperpolarizing and depolarizing current |
| Recovery cycle (RC) | Recovery of excitability and evaluates parameters such as refractoriness, subnormality and supernormality by giving paired stimuli between different interstimulus intervals. |
